# Increased radiographic progression of distal hand osteoarthritis occurring during biologic DMARD monotherapy for concomitant rheumatoid arthritis

**DOI:** 10.1186/s13075-021-02654-0

**Published:** 2021-10-26

**Authors:** C. A. Lechtenboehmer, T. Burkard, S. Reichenbach, U. A. Walker, A. M. Burden, T. Hügle

**Affiliations:** 1 Department of Radiology, University Hospital of Basel, Basel, Switzerland; 2 Department of Chemistry and Applied Biosciences, Institute of Pharmaceutical Sciences, ETH Zurich, Zurich, Switzerland; 3 Institute for Social and Preventive Medicine, University of Bern, Bern, Switzerland; 4 Department of Rheumatology, Immunology, and Allergology, Bern University Hospital, Bern, Switzerland; 5 Department of Rheumatology, University Hospital of Basel, Basel, Switzerland; 6 Department of Rheumatology, Lausanne University Hospital (CHUV) and University of Lausanne, Av. Pierre-Decker 4, 1011 Lausanne, Switzerland

## Abstract

**Objectives:**

A considerable proportion of patients with rheumatoid arthritis (RA) also suffer from hand osteoarthritis (OA). We here assess the association between conventional synthetic (cs) and biological (b) disease-modifying antirheumatic drugs (DMARDs) and radiographic distal interphalangeal-(DIP) OA in patients with RA.

**Methods:**

Adult RA patients from a longitudinal Swiss registry of rheumatic diseases who had ≥ 2 hand radiographs were included at the first radiograph and followed until the outcome or the last radiograph. Patients were grouped into two cohorts based on whether DIP OA was present or absent at cohort entry (cohorts 1 and 2, respectively). Modified Kellgren-Lawrence scores (KLS) were obtained by evaluating DIP joints for the severity of osteophytes, joint space narrowing, subchondral sclerosis, and erosions. KLS ≥ 2 in ≥ 1 DIP joint indicated incident or existing OA, and increase of ≥ 1 in KLS in ≥ 1 DIP joint indicated progression in existing DIP OA. Time-varying Cox regression and generalized estimating equation (GEE) analyses were performed. We estimated hazard ratios (HRs) and odds ratios (ORs) with 95% confidence intervals (CI) of DIP OA incidence (cohort 2), or progression (cohort 1), in bDMARD monotherapy, bDMARD/csDMARD combination therapy, and past or never DMARD use, when compared to csDMARD use. In post hoc analyses, we descriptively and analytically assessed the individual KLS features in cohort 1.

**Results:**

Among 2234 RA patients with 5928 radiographs, 1340 patients had DIP OA at baseline (cohort 1). Radiographic progression of DIP OA was characterized by new or progressive osteophyte formation (666, 52.4%), joint space narrowing (379, 27.5%), subchondral sclerosis (238, 17.8%), or erosions (62, 4.3%). bDMARD monotherapy had an increased risk of radiographic DIP OA progression compared to csDMARD monotherapy (adjusted HR 1.34 [95% CI 1.07–1.69]). The risk was not significant in csDMARD/bDMARD combination users (HR 1.12 [95% CI 0.96–1.31]), absent in past DMARD users (HR 0.96 [95% CI 0.66–1.41]), and significantly lower among never DMARD users (HR 0.54 [95% CI 0.33–0.90]). Osteophyte progression (HR 1.74 [95% CI 1.11–2.74]) was the most significantly increased OA feature with bDMARD use compared to csDMARD use. In 894 patients without initial DIP OA (cohort 2), the risk of incident OA did not differ between the treatment groups. The results from GEE analyses corroborated all findings.

**Conclusions:**

These real-world RA cohort data indicate that monotherapy with bDMARDs is associated with increased radiographic progression of existing DIP OA, but not with incident DIP OA.

**Supplementary Information:**

The online version contains supplementary material available at 10.1186/s13075-021-02654-0.

## What is already known about this subject?

- There are conflicting data about the benefit of treatment with cs- or bDMARDs for distal hand osteoarthritis.
- Direct and indirect osteo-anabolic properties of DMARDs are known, but the net effect on osteophytosis as a hallmark of DIP OA is unclear.

## What does this study add?

- bDMARD monotherapy enhanced pre-existing distal hand osteoarthritis compared to csDMARD standard therapy in a predominantly female and postmenopausal RA population.
- Growing of osteophytes is the main underlying cause of increased radiographic progression occurring under bDMARDs.
- Concomitant osteoporosis or receiving osteoporosis therapy reduced this effect.

## How might this impact clinical practice or future developments?

- Hand radiographs of RA patients at baseline and during follow-up undergoing DMARD therapy should be assessed for distal HOA lesions.
- In patients with pre-existing DIP OA undergoing bDMARD monotherapy, the hands should be monitored radiographically.

## Key messages

- Biological DMARD monotherapy potentially enhances the risk of progression of pre-existing DIP OA in RA patients, mainly through osteophyte growth.
- This effect is stronger under non-TNFi bDMARDs and in patients under 55 years of age.
- There is no increased incidence of DIP OA with cs- or bDMARD therapy in patients with RA.
- Osteoporosis or anti-osteoporotic therapy, but not prednisone, diminishes the risk of radiographic DIP OA progression under bDMARDs.

## Introduction

Hand osteoarthritis (HOA) and rheumatoid arthritis (RA) are two distinct entities, which often occur simultaneously. Marginal erosions are a radiologic hallmark of RA, while HOA is typically characterized by osteophyte formation, usually in the distal (DIP) or proximal interphalangeal (PIP) joints [1]. Radiographic progression of HOA is a dynamic process. In contrast to marginal erosions, which can be observed in the early stages of RA, central erosions in OA usually occur after joint space collapse. In DIP OA, the joint space undergoes radiographic reorganization, known as the “repair phase,” a finding that typically does not occur in RA joints [2].

Inflammation seems to be a driver of DIP OA. Treatment with prednisone over 6 weeks has been reported to effectively reduce pain in inflammatory HOA [3]. Moreover, in a cohort of patients with concomitant RA, prolonged systemic inflammation with a high erythrocyte sedimentation rate (esr) over 3 years was suggested to be a risk factor for radiographic DIP OA incidence [4]. In contrast, in a previous study, we found no association between RA disease activity measured by DAS28-BSR (RA disease activity score using 28 joints and esr) and radiographic progression of DIP OA [5]. There is a multitude of efficacious RA medications, some of those have been studied also in HOA. The use of methotrexate over 3 months in patients with erosive HOA was associated with a significantly increased rate of radiographic transformation from the erosive to the repair phase, indicating a potential anti-erosive effect of methotrexate in HOA [6]. However, data on TNF inhibitors (TNFis) in HOA are controversial. Treatment with etanercept or adalimumab was associated with increased radiographic subchondral remodeling after 12 months and anti-erosive effects by TNFi were described in inflammatory DIP OA [7, 8]. In another 12-week prospective trial, adalimumab had no effect on MRI-detected synovitis or bone marrow lesions in HOA [9]. In patients with concomitant RA, infliximab mitigated the progression of HOA in the PIP but not in DIP joints [4], potentially because the DIP joints are rarely affected by RA. On the other hand, Loef et al. described a reduced risk of radiographic progression of DIP OA in 143 patients undergoing prolonged TNFi-methotrexate combination therapy in an RA cohort [10].

The impact of conventional synthetic (cs) or biological (b) DMARDs on radiographic HOA as mono- versus combination therapy has never been studied over the long term. This is of interest as DMARDs reduce bone resorption and, as consequence, stimulate new bone with a potential negative impact on osteophytes. Thus, we aimed to assess the association between several DMARDs and DIP OA in RA patients given a 17-year study period.

## Methods

### Study design and data source

We conducted a cohort study using data derived from patients in the Swiss Clinical Quality Management in Rheumatic Diseases (SCQM) registry. The SCQM registry was established in 1997 and is used to prospectively follow RA patients [11]. RA diagnoses are made by board-certified rheumatologists. Follow-up for the SCQM registry involves annual physical examination (i.e., tender joint count, swollen joint count), disease activity scores (e.g., DAS28), laboratory tests (i.e., esr), and radiographs. Other relevant health issues such as diagnosis of osteoporosis and its treatment are reported by the patient and recorded by the rheumatologist into SCQM. Clinical information is usually updated every time a patient changes anti-rheumatic therapy but at least once a year during a regular visit. Ethical approval for this study was obtained from the local ethics committees. All patients provided written informed consent.

### Study population

We included patients with clinically diagnosed RA who had at least two eligible hand radiographs taken between January 1997 and October 2014. Radiographs were considered eligible if all eight DIP joints could be scored. Patients entered the study on the date of their first eligible radiograph. Each individual’s observation period lasted until the outcome or the final eligible radiograph. We divided the study population into two cohorts based on whether DIP OA (assessed by modified KLS) was present or absent at cohort entry (cohorts 1 and 2, respectively).

### Exposures

We defined the following mutually exclusive treatment groups: csDMARD monotherapy (comparator group given the biggest size), bDMARD monotherapy, cs/bDMARD combination therapy, past use of any DMARD, and previously unexposed to DMARD therapy (called never-use). csDMARDs included methotrexate, leflunomide, sulfasalazine, and chloroquine. bDMARDs included TNFis (e.g., adalimumab, certolizumab, etanercept, infliximab, and golimumab) and non-TNFis (e.g., abatacept, tocilizumab and rituximab), by which we additionally stratified in cohort 2 given the observed increased risks of DIP OA progression of bDMARD users. The targeted synthetic DMARD tofacitinib was available during the observation period but was not used by any of the eligible patients.

The current exposure included 31 days after the end of supply. Thus, past users were defined as having been off treatment for at least this period.

Exposures of interest were assessed at baseline for time-invariant analyses and additionally at every other radiograph or visit for time-varying analyses. The date of the radiographic evaluation and visit date coincided in 88.8% of cases, and when we allowed a 1-month time window to assign radiographic information to the patient’s visit, this number increased to 93.3% (i.e., 6.7% of radiographs did not have accompanying visit information). Patient visits were recorded in SCQM on an average of once per year, and this interval was similar between the treatment groups (Additional file 1: Table S1). In time-varying analyses, we accounted for patients changing exposure groups during their observation period. Examples of exposure evaluation for time-varying analyses can be seen in Additional file 1: Figure S1.

### Outcome

A trained radiology resident (CAL) blinded to patient data assessed conventional postero-anterior hand radiographs from each patient with known time order. The reader was trained by a set of 100 radiographs that have been evaluated by two senior rheumatologists (TH, UAW). As the standard for reading DIP OA, we used the Osteoarthritis Research Society International (OARSI) atlas [12]. DIP joints were scored for osteophyte severity (range 0–3), joint space narrowing (range 0–3), the presence of subchondral sclerosis, and the presence of central erosions, and these evaluations were used to formulate the modified KLS (range 0–4) [13].

Following the initial assessment of 7499 hand radiographs from 2870 RA patients, groups of 200 radiographs with an average of 60 patients were reassessed until Cohen’s *κ* coefficient, an indicator of intra-rater reliability, reached the pre-defined value of *κ* ≥ 0.70 [14]. This value was exceeded after 800 radiographs were assessed. The re-evaluated scores were used for the final analysis. In the final 200 rescored radiographs, kappa values were recorded as follows: K/L grade 0.90, osteophyte severity 0.77, joint space narrowing 0.83, subchondral sclerosis 0.91, and central erosions 0.92. The percentage of erosive joint surface destruction (in 19% increments) of bilateral metacarpophalangeal (MCP) joints 2–5 (with an unknown chronology) was assessed by the SCQM foundation using a similar scoring method to that described by Rau et al. In brief, the Ratingen score is based on the amount of joint surface destruction of each MCP and PIP joint. For this analysis, percentages of erosive joint surface destruction in all 8 MCP joints were summed (range 0–800%) and categorized. The intra-class correlation coefficient was 0.98.

Our primary outcome, assessed in cohort 1, was OA progression defined as an increase of ≥ 1 point in the summed KLSs of all eight DIP joints (range 0–32). The reliability of change was estimated by the *κ* value of 0.84; the smallest detectable change was a KLS of 0.13.

Our secondary outcome, assessed in cohort 2, was the rate of incident OA defined as a KLS ≥ 2 in ≥ 1 DIP joint (K/L grade 1 [i.e., questionable OA] was not rated as OA).

### Covariates

Based on the literature review, we included the following a priori covariates in our adjusted analyses since they are confounders or risk factors for incident/progressive HOA: age (continuous), sex (binary), body mass index (BMI, continuous), RA duration (continuous), rheumatoid factor (RF) status (binary), DAS28-esr score (continuous), prednisone use (binary), cardiac disorders (i.e., myocardial infarction, ischemic heart disease, congestive heart failure, binary), hypertension (diagnosis or treatment, binary), osteoporosis (diagnosis or treatment, binary), hand surgery (binary), or large joint OA or hip/knee arthroplasty (binary). All variables were assessed at each radiograph/visit except for sex and RF status, which were given per patient (i.e., their values could not change over time).

In time-invariant analyses (baseline model), we performed a sensitivity analysis in which we additionally adjusted for the use of csDMARDs for ≥ 1 year prior to cohort entry and the use of bDMARDs for ≥ 1 year prior to cohort entry to account for prevalent use of cs/bDMARDs at cohort entry. This analysis yielded slightly higher HRs of DIP OA incidence/progression in bDMARD and cs/bDMARD combination therapy users than did the overall baseline analysis, data not shown.

A total of four variables had missing data (BMI, RA duration, RF status, DAS28-esr score). All variables for which we aimed to adjust for as well as the exposure and the outcome variable were part of the model with which we performed the two-level multiple imputation (by patient) of the missing values. We used the fully conditional specification imputation method and the Gibbs Markov chain Monte Carlo algorithm (because of some small clusters) in the BLIMP software 2.2 [15]. Although the overall missingness was only around 20%, we imputed 40 datasets. The imputation model was tested using the potential scale reduction (PSR) and performed at a PSR of < 1.02 which indicates model convergence. Detailed information about data missingness and the multiple imputation method can be found in Additional file 1: Tables S2–S4.

### Statistical analysis

Statistical analyses were performed separately for the two cohorts 1 and 2. We described patient characteristics per exposure group at cohort entry (i.e., information changing over time as used in time-varying analyses was not described). Using Cox proportional hazard (PH) regression analysis, we estimated crude and adjusted hazard ratios (HRs) and 95% confidence intervals (CI) of DIP OA progression (cohort 1) and of incident DIP OA (cohort 2) using patient information at cohort entry only in all exposure groups (i.e., bDMARD, cs/bDMARD combination, past DMARD use, never DMARD use) when compared to csDMARD use (called baseline model). Cox PH assumptions were tested using the Martingale residual method and did not hold for csDMARD use or hypertension in cohort 1, or for never DMARD use in cohort 2. Therefore, since exposure and covariates changed over time, we additionally used time-varying Cox regression analyses. We additionally estimated the crude incidence rates as absolute risks based on the numbers of events and determined the follow-up times per exposure group. To test the model specifications, we chose at random one imputed dataset. The lowest Akaike Information Criterion (AIC) was reached with linear terms (i.e., introduction of interaction terms, quadratic or cubic terms did not lower the AIC value). Furthermore, for robustness assessment, we repeated our overall time-varying analyses using generalized estimating equation (GEE) analysis. Examples of data management for each analysis (Cox PH regression analysis, time-varying Cox regression analysis, GEE analysis) can be seen in Additional file 1: Tables S5–S7.

In exploratory subgroup analyses, we assessed subgroups of age (≤ 55 years, > 55 years) and RF status (positive, negative) at cohort entry. Moreover, because of their potential influence on bone turnover, we assessed subgroups of concomitant osteoporosis and concurrent prednisone in time-varying analyses. Since we aimed to assess the impact of concomitant prednisone and bDMARD use, *bDMARD use without prednisone use* was the comparator in this subgroup analysis.

DAS28-esr may be seen as a mediator variable; therefore, we performed a sensitivity analysis without adjusting for it in time-varying Cox regression analyses. Furthermore, since the detection of the outcome is only possible if a radiograph is taken, in sensitivity analyses using time-varying Cox regression, we led the outcome by 6 months (183 days).

Given the observed increased risks of DIP OA progression of bDMARD users, in a post hoc analysis in cohort 1, we descriptively and analytically assessed the progression of individual KLS components (osteophytes, joint space narrowing, sclerosis, and erosion). Progression of osteophyte and joint space narrowing were defined by an increase of ≥ 1 score in ≥ 1 DIP (continuous measurement). Sclerosis and erosion were measured binary, and progression was defined as an increase of ≥ 1 in frequency. In a further post hoc analysis, we adjusted our Cox regression analyses of DIP OA for fewer variables (i.e., age, sex, rheumatoid arthritis duration, hypertension, osteoporosis) because some of the smaller exposure groups ran the risk of overfitting.

The GEE analyses were performed in Stata/IC 16 while all other analyses were performed using the SAS statistical software version 9.4 (NC, USA).

## Results

### Baseline characteristics

**Fig. 1:**
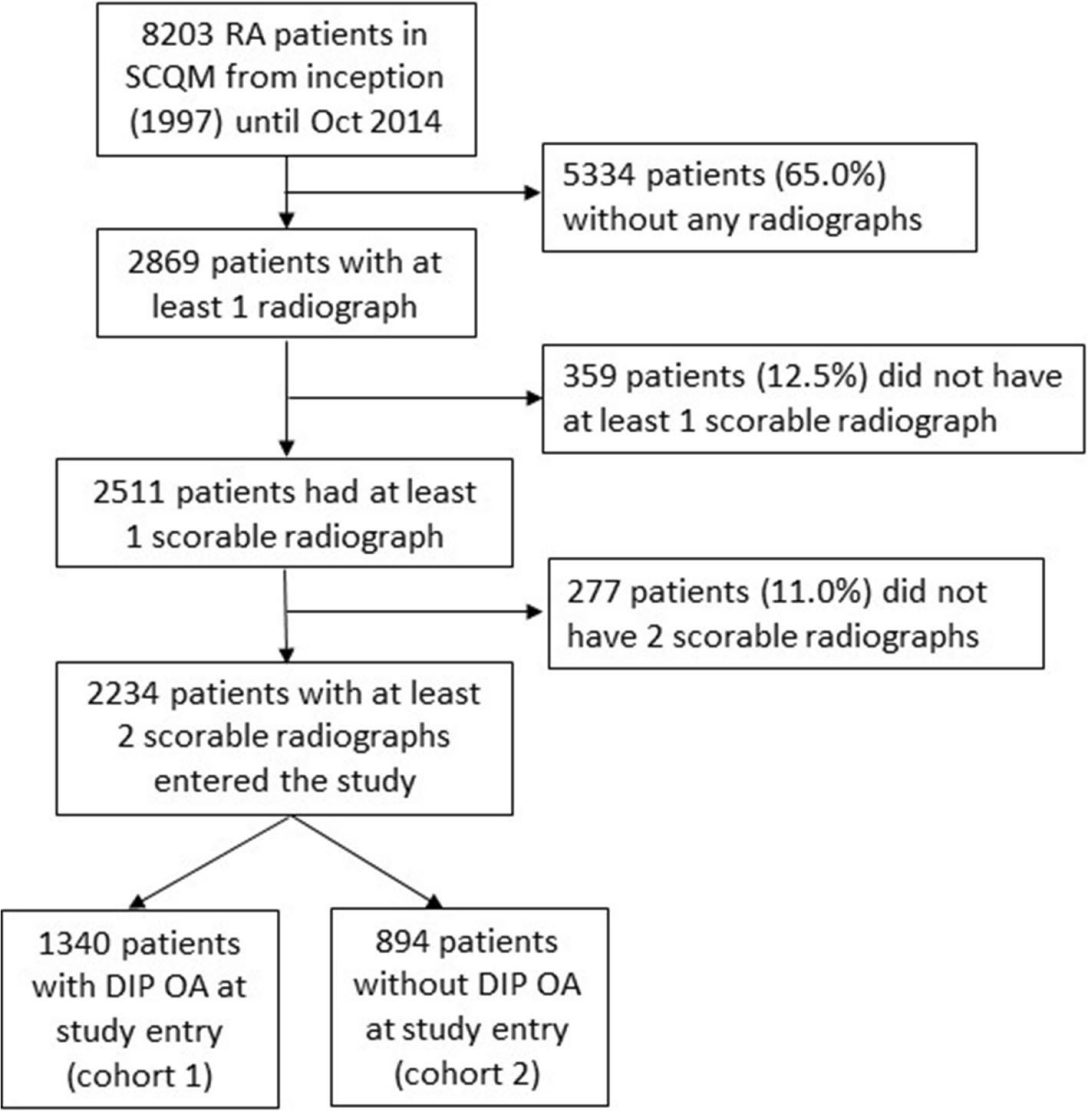
Flow chart of the study population. DIP, distal interphalangeal; OA, osteoarthritis; SCQM, Swiss Clinical Quality Management in Rheumatic Diseases

A total of 8203 RA patients are included in SCQM until October 2014. Thereof, 2869 patients had at least 1 radiograph taken. A total of 2234 patients with at least two scorable hand radiographs were eligible for this study (Fig. 1), and the patients are provided in Additional file 1: Table S8.

A total of 1340 patients had DIP OA at baseline and were included in the analysis of progression. The majority of patients were receiving csDMARDs (*n* = 847), bDMARDs (*n* = 72), or combination therapy (*n* = 257), while the remainder had used DMARDs in the past (*n* = 19) or were previously unexposed to DMARD therapy (*n* = 145). Every patient had on average 2.7 radiographs taken with a median of 3.0 years (interquartile range 2.0–4.4 years) between two radiographs. The median duration between the two radiographs in cohort 1 ranged between 2.4 years in never DMARD users and 3.2 years in bDMARD users or past DMARD users and was generally higher in patients in cohort 2 without DIP OA (Additional file 1: Table S9).

Table 1 presents the patient information at baseline. Around 77% of the patients were female, with a slightly higher proportion of women in the bDMARD group (83.3%). The mean age was also similar between the exposure groups, ranging from 58 to 61 years. The mean duration of follow-up ranged from 2.8 years in past users to 4.3 years in never-users. The mean DAS28-esr was 4.0, with similar standard deviations of around 1.4 in patients with csDMARD or bDMARD monotherapy. The percentage of patients who used additional prednisone was approximately 52% in the csDMARD, bDMARD, and past user groups; 57.2% in the combination therapy group; and 42.8% in the never-user group. Compared to the csDMARD group, the bDMARD group, but not the past- and never-user groups, had longer RA disease duration, higher proportions of rheumatoid factor positivity, hypertension, cardiac disorders, and osteoporosis.

**Table 1:** Patient characteristics of patients with DIP OA at cohort entry

Patient characteristics at baseline visit	csDMARD, *n* = 847	bDMARD, *n* = 72	Combination, *n* = 257	Past-use, *n* = 19	Never-use, *n* = 145
Mean age [years] (SD)	60.1 (9.9)	61.9 (10.5)	58.6 (10.6)	60.7 (9.9)	60.9 (10.1)
Female (%)	653 (77.1%)	60 (83.3%)	195 (75.9%)	13 (68.4%)	112 (77.2%)
Mean follow-up time [years] (SD)	3.9 (2.3)	3.4 (1.6)	3.4 (1.9)	2.8 (1.2)	4.3 (2.8)
Mean BMI (SD)	25.9 (4.9)	24.9 (4.0)	25.6 (5.8)	25.6 (2.3)	25.6 (4.5)
Missing (%)	152 (17.9%)	3 (4.2%)	9 (3.4%)	6 (31.6%)	58 (40.0%)
Median RA duration [years] (IQR)	5.3 (2.0–12.8)	11.2 (5.0–18.9)	8.0 (3.9–14.4)	6.5 (2.7–8.7)	6.9 (1.7–16.4)
Missing (%)	21 (2.5%)	3 (4.2%)	8 (3.0%)	1 (5.3%)	7 (4.8%)
RF negative (%)	275 (32.5%)	33 (45.8%)	77 (30.0%)	6 (31.6%)	53 (36.6%)
RF positive (%)	559 (66.0%)	36 (50.0%)	163 (63.4%)	10 (52.6%)	78 (53.8%)
Missing (%)	13 (1.5%)	3 (4.2%)	17 (6.6%)	3 (15.8%)	14 (9.7%)
Mean DAS28-esr^a^ (SD)	4.0 (1.5)	4.1 (1.3)	3.9 (1.5)	3.7 (1.8)	4.4 (1.4)
Missing (%)	44 (5.2%)	1 (1.4%)	13 (5.1%)	4 (21.1%)	37 (25.6%)
≥ 365 days of current csDMARD use^b^ (%)	281 (33.2%)	0	129 (50.2%)	0	0
≥ 365 days of current bDMARD use^c^ (%)	0	18 (25.0%)	56 (21.8%)	0	0
Prednisone use^d^ (%)	443 (52.3%)	37 (51.4%)	147 (57.2%)	10 (52.6%)	62 (42.8%)
Cardiac disorders^e^ (%)	80 (9.5%)	12 (16.7%)	25 (9.7%)	7 (36.8%)	10 (6.9%)
Hypertension^f^ (%)	206 (24.3%)	27 (37.5%)	52 (20.2%)	4 (21.1%)	37 (25.5%)
Osteoporosis or fracture^g^ (%)	106 (12.5%)	27 (37.5%)	76 (29.6%)	1 (5.3%)	16 (11.0%)
Large joint osteoarthritis^h^ (%)	82 (9.7%)	9 (12.5%)	45 (17.5%)	5 (26.3%)	17 (11.7%)
Hand surgery (%)	67 (7.9%)	15 (20.8%)	33 (12.8%)	1 (5.3%)	12 (8.3%)

*DIP OA* distal interphalangeal joint osteoarthritis, *bDMARD* biologic disease-modifying antirheumatic drug, *BMI* body mass index, *csDMARD* conventional synthetic disease-modifying antirheumatic drug, *DAS* Disease Activity Score, *esr* erythrocyte sedimentation rate, *IQR* interquartile range, *RA* rheumatoid arthritis, *RF* rheumatoid factor, *SD* standard deviation

^a^DAS28-esr = (0.56 × √[TJC28] + 0.28 × √[SJC28] + 0.70 × ln[ESR]) × 1.08 + 0.16

^b^csDMARD use includes methotrexate, leflunomid, sulfasalazin, chloroquine, azathioprine, cyclosporin A, and cyclophosphamid

^c^bDMARD use includes abatacept, adalimumab, anakinra, certolizumab, etanercept, golimumab, infliximab, rituximab, and tocilizumab

^d^Prednisone use includes systemic or intra-articular prednisone use

^e^Cardiac disorders include angina pectoris, myocardial infarction, arrhythmias, ischemic heart failure, angioplasty, or their treatment

^f^Hypertension includes also anti-hypertensive treatment

^g^Osteoporosis or fractures include also anti-osteoporotic treatment

^h^Large joint osteoarthritis includes hip/knee replacements

### Effects of DMARDs on radiographic DIP OA evolution

Among the 1340 patients with radiographic DIP OA at cohort entry, time-varying Cox analyses demonstrated a significantly higher HR for radiographic DIP OA progression in patients receiving bDMARD (1.34 [95% CI 1.07–1.69]) versus csDMARD monotherapy. Conversely, HRs were lower in past DMARD users (HR 0.96 [95% CI 0.66–1.41]) and never DMARD users (HR 0.54 [95% CI 0.33–0.90]) [Table 2]. The latter indicates a 46% lower risk of DIP OA progression in DMARD-naive RA patients compared to the csDMARD group.

**Table 2:** Incidence rates, hazard ratios, and odds ratios of hand OA progression per treatment group following Cox regression analysis and GEE analysis among those with distal interphalangeal osteoarthritis at baseline (*N* = 1340)

Exposure	Hand OA progression events	Person-time [years]	IR per 1000 person-years	Crude HR (95% CI)	Adjusted HR (95% CI)^a^	Crude OR (95% CI)	Adjusted OR (95% CI)^a^
Analysis using baseline information only							
csDMARD	526	3306.0	159.1	Ref (1.00)	Ref (1.00)	NA	NA
bDMARD	34	244.7	138.9	0.96 (0.71–1.31)	1.07 (0.78–1.46)	NA	NA
Combination	132	886.6	148.9	1.03 (0.85–1.24)	1.09 (0.89–1.32)	NA	NA
Past-use	8	53.8	148.7	1.24 (0.70–2.19)	1.15 (0.60–2.20)	NA	NA
Never-use	83	629.7	131.8	0.77 (0.61–0.97)	0.81 (0.63–1.02)	NA	NA
Time-varying analysis							
csDMARD	421	2885.7	145.9	Ref (1.00)	Ref (1.00)	Ref (1.00)	Ref (1.00)
bDMARD	79	391.9	201.6	1.23 (0.98–1.54)	1.34 (1.07–1.69)	1.49 (1.15–1.93)	1.53 (1.17–2.00)
TNFi	67	357.6	187.4	1.16 (0.91–1.48)	1.26 (0.98–1.62)	1.36 (1.03–1.79)	1.41 (1.06–1.88)
Non-TNFi	12	34.3	349.9	1.85 (1.18–2.89)	2.07 (1.35–3.20)	2.94 (1.50–5.77)	2.79 (1.41–5.52)
Combination	236	1378.7	171.2	1.05 (0.90–1.22)	1.12 (0.96–1.31)	1.12 (0.95–1.33)	1.16 (0.97–1.38)
TNFi	210	1293.7	162.3	1.01 (0.86–1.18)	1.09 (0.92–1.28)	1.07 (0.90–1.28)	1.11 (0.93–1.33)
Non-TNFi	26	85	305.9	1.54 (1.10–2.15)	1.56 (1.10–2.23)	1.75 (1.13–2.73)	1.77 (1.13–2.77)
Past-use	24	128.4	186.9	0.96 (0.64–1.42)	0.96 (0.66–1.41)	1.36 (0.87–2.14)	1.30 (0.83–2.05)
Never-use	23	335.9	68.5	0.51 (0.31–0.83)	0.54 (0.33–0.90)	0.89 (0.57–1.38)	0.91 (0.58–1.42)

*bDMARD* biologic disease-modifying antirheumatic drug, *CI* confidence interval, *csDMARD* conventional synthetic disease-modifying antirheumatic drug, *IR* incidence rate, *HR* hazard ratio, *OA* osteoarthritis, *OR* odds ratio, *TNFi* tumor necrosis factor inhibitor

^a^Adjusted for age, sex (time-invariant), body mass index, rheumatoid arthritis duration, rheumatoid factor (time-invariant), DAS28-esr score, prednisone use, cardiac disorders, hypertension, osteoporosis, hand surgery, large joint osteoarthritis, or hip/knee arthroplasty

CsDMARD/bDMARD combination therapy showed no significant difference compared to csDMARD monotherapy (1.12 [95% CI 0.96–1.31]). Regarding bDMARD monotherapy, the effect size of DIP OA progression was higher in the non-TNFi subgroup (2.07 [95% CI 1.35–3.20]) than in the TNFi subgroup (1.26 [95% CI 0.98–1.62]), though the number of events in the former group was small.

In the explorative subgroup analyses, we observed higher effect sizes for radiographic DIP OA progression under bDMARD therapy in patients ≤ 55 years (1.49 [95% CI 1.01–2.21]) than in those > 55 years (1.21 [95% CI 0.90–1.61]) [Table 3]. In both the csDMARD and bDMARD monotherapy groups, there was a lower risk of DIP OA progression in patients with concomitant osteoporosis, or osteoporosis therapy, than in those without. Conversely, neither rheumatoid factor positivity nor prednisone therapy was associated with DIP OA progression under either bDMARD or combination therapy.

**Table 3:** Hazard ratios of hand OA progression per treatment group following time-varying Cox regression analyses in subgroups based on age, rheumatoid factor, osteoporosis (treatment), and prednisone use

Exposure	Hand OA progression events	Person-time [years]	Crude HR (95% CI)	Adjusted HR (95% CI)^a^
Age ≤ 55 years at cohort entry				
csDMARD	130	860.6	Ref (1.00)	Ref (1.00)
bDMARD	31	127.4	1.36 (0.93–2.00)	1.49 (1.01–2.21)
Combination	87	526.8	0.94 (0.73–1.22)	1.01 (0.78–1.33)
Past-use	10	39.2	0.96 (0.41–2.26)	1.09 (0.51–2.34)
Never-use	6	111.3	0.34 (0.14–0.80)	0.34 (0.14–0.84)
Age > 55 years at cohort entry				
csDMARD	291	2025.1	Ref (1.00)	Ref (1.00)
bDMARD	48	264.6	1.13 (0.86–1.51)	1.21 (0.90–1.61)
Combination	149	851.9	1.11 (0.92–1.33)	1.16 (0.96–1.41)
Past-use	14	89.2	0.84 (0.52–1.36)	0.79 (0.48–1.29)
Never-use	17	224.6	0.62 (0.35–1.10)	0.68 (0.37–1.25)
Rheumatoid factor negative at cohort entry				
csDMARD	132	1014.4	Ref (1.00)	Ref (1.00)
bDMARD	28	167.5	1.22 (0.83–1.80)	1.33 (0.90–1.96)
Combination	83	503.8	1.11 (0.85–1.44)	1.21 (0.92–1.58)
Past-use	7	35.3	1.21 (0.61–2.38)	1.16 (0.58–2.33)
Never-use	6	126.8	0.42 (0.19–0.92)	0.45 (0.20–1.03)
Rheumatoid factor positive at cohort entry				
csDMARD	284	1832.4	Ref (1.00)	Ref (1.00)
bDMARD	45	210.7	1.20 (0.89–1.60)	1.40 (1.04–1.89)
Combination	139	809.4	0.99 (0.82–1.21)	1.09 (0.89–1.32)
Past-use	17	86.6	0.87 (0.52–1.42)	0.86 (0.55–1.36)
Never-use	16	186.2	0.58 (0.31–1.10)	0.59 (0.31–1.13)
Osteoporosis/fracture or osteoporosis treatment (additionally time-varying)				
csDMARD without osteoporosis	334	2342.2	Ref (1.00)	Ref (1.00)
csDMARD with osteoporosis	87	543.5	0.88 (0.69–1.13)	0.87 (0.68–1.11)
bDMARD without osteoporosis	50	226.5	1.35 (1.04–1.77)	1.43 (1.09–1.88)
bDMARD with osteoporosis	29	165.4	1.00 (0.70–1.43)	1.04 (0.72–1.49)
Combination therapy without osteoporosis	165	952.5	1.06 (0.89–1.27)	1.13 (0.94–1.35)
Combination therapy with osteoporosis	71	426.2	0.94 (0.75–1.18)	0.95 (0.76–1.20)
Never/past-use without osteoporosis	38	390.3	0.65 (0.44–0.95)	0.69 (0.47–1.02)
Never/past-use with osteoporosis	9	74.0	0.68 (0.34–1.37)	0.66 (0.34–1.29)
Prednisone treatment (additionally time-varying)				
csDMARD without prednisone	219	1509.1	0.79 (0.57–1.08)	0.72 (0.52–1.00)
csDMARD with prednisone	202	1376.6	0.87 (0.64–1.20)	0.81 (0.59–1.12)
bDMARD without prednisone	38	183.8	Ref (1.00)	Ref (1.00)
bDMARD with prednisone	41	208.2	1.03 (0.69–1.54)	1.07 (0.72–1.60)
Combination therapy without prednisone	128	679.8	0.90 (0.64–1.25)	0.88 (0.63–1.22)
Combination therapy with prednisone	108	699.0	0.83 (0.60–1.17)	0.83 (0.59–1.17)
Never/past-use without prednisone	22	287.8	0.39 (0.23–0.67)	0.36 (0.21–0.63)
Never/past-use with prednisone	25	176.5	0.90 (0.54–1.47)	0.87 (0.53–1.45)

*bDMARD* biologic disease-modifying antirheumatic drug, *CI* confidence interval, *csDMARD* conventional synthetic disease-modifying antirheumatic drug, *HR* hazard ratio

^a^Adjusted for age, sex (time-invariant), body mass index, rheumatoid arthritis duration, rheumatoid factor (time-invariant), DAS28-esr score, prednisone use, cardiac disorders, hypertension, osteoporosis, hand surgery, large-joint osteoarthritis, or hip/knee arthroplasty

Table 4 presents the post hoc analyses of the KLS components. We observed that 52.4% (666 progressions) of patients progressed in terms of new osteophyte formation, 27.5% (379 progressions) in joint space narrowing, 17.8% (238 progressions) in sclerosis, and 4.3% in erosion (62 progressions). The time-varying Cox regression analysis yielded the highest HRs of osteophyte progression (1.74 [95% CI 1.11–2.74]), joint space narrowing (1.38 [95%CI 1.01–1.86]) in bDMARD users compared to csDMARD users. Conversely, the HR for erosions was not statistically significant (1.51 [95%CI 0.76–3.00]). Additional file 1: Table S10 presents the post hoc analyses when adjusting the Cox regression analyses of DIP OA progression for fewer variables; the results remained unchanged.

**Table 4:** Hazard ratios of progression in osteoarthritis components (osteophytes, joint space narrowing, sclerosis, and erosions) per treatment group in time-varying Cox regression analysis

Exposure	**Osteophyte progression events**	**Person-time [years]**	**Crude HR (95% CI)**	**Adjusted HR (95% CI)**^**a**^
csDMARD	404	2883.3	Ref (1.00)	Ref (1.00)
bDMARD	77	400.3	1.56 (0.90–2.47)	1.74 (1.11–2.74)
TNFi	66	362.7	1.16 (0.90–1.48)	1.25 (0.97–1.62)
Non-TNFi	11	37.3	1.60 (1.01–2.53)	1.82 (1.15–2.87)
Combination	226	1424.9	0.96 (0.82–1.13)	1.02 (0.87–1.20)
TNFi	203	1321.9	0.98 (0.83–1.16)	1.06 (0.90–1.25)
Non-TNFi	23	103	1.08 (0.73–1.61)	1.16 (0.78–1.71)
Past-use	23	131.5	0.90 (0.61–1.35)	0.91 (0.61–1.36)
Never-use	18	337.5	0.40 (0.23–0.68)	0.41 (0.23–0.72)
Exposure	**JSN progression events**	**Person-time [years]**	**Crude HR (95% CI)**	**Adjusted HR (95% CI)**^**a**^
csDMARD	231	3239.2	Ref (1.00)	Ref (1.00)
bDMARD	51	458.1	1.80 (0.92–3.49)	1.38 (1.01–1.86)
TNFi	43	410.4	1.33 (0.97–1.82)	1.32 (0.95–1.83)
Non-TNFi	8	47.4	1.88 (0.96–3.66)	1.76 (0.89–3.46)
Combination	136	1636.7	0.98 (0.79–1.20)	1.04 (0.83–1.29)
TNFi	124	1531	1.02 (0.82–1.27)	1.03 (0.82–1.29)
Non-TNFi	12	105.8	1.15 (0.67–1.98)	1.12 (0.65–1.94)
Past-use	16	145.8	1.15 (0.72–1.83)	1.19 (0.74–1.92)
Never-use	15	343.6	0.71 (0.40–1.27)	0.83 (0.46–1.48)
Exposure	**Sclerosis progression events**	**Person-time [years]**	**Crude HR (95% CI)**	**Adjusted HR (95% CI)**^**a**^
csDMARD	160	3302.2	Ref (1.00)	Ref (1.00)
bDMARD	27	484.1	1.66 (0.77–3.57)	1.07 (0.72–1.60)
TNFi	22	432.3	0.95 (0.61–1.46)	0.97 (0.63–1.52)
Non-TNFi	5	51.8	1.67 (0.77–3.60)	1.89 (0.87–4.11)
Combination	84	1673.7	0.90 (0.69–1.17)	0.97 (0.74–1.27)
TNFi	75	1554.2	0.90 (0.69–1.18)	0.94 (0.71–1.24)
Non-TNFi	9	119.5	1.28 (0.67–2.45)	1.34 (0.71–2.52)
Past-use	10	159.5	1.02 (0.53–1.94)	1.04 (0.53–2.01)
Never-use	9	344.7	0.67 (0.34–1.35)	0.71 (0.35–1.45)
Exposure	**Erosion progression**	**Person-time [years]**	**Crude HR (95% CI)**	**Adjusted HR (95% CI)**^**a**^
csDMARD	41	3571.5	Ref (1.00)	Ref (1.00)
bDMARD	12	542.5	2.77 (0.79–9.70)	1.51 (0.76–3.00)
TNFi	9	481.1	1.46 (0.71–3.00)	1.32 (0.62–2.80)
Non-TNFi	NA^b^	NA^b^	NA^b^	NA^b^
Combination	19	1857.3	0.72 (0.42–1.25)	0.78 (0.43–1.40)
TNFi	17	1717.3	0.76 (0.43–1.34)	0.76 (0.41–1.39)
Non-TNFi	NA^b^	NA^b^	NA^b^	NA^b^
Past-use	6	182.7	2.35 (1.05–5.26)	2.40 (1.04–5.56)
Never-use	NA^b^	NA^b^	NA^b^	NA^b^

*bDMARD* biologic disease-modifying antirheumatic drug, *CI* confidence interval, *csDMARD* conventional synthetic disease-modifying antirheumatic drug, *IR* incidence rate, *HR* hazard ratio, *OA* osteoarthritis, *OR* odds ratio, *TNFi* tumor necrosis factor inhibitor, *JSN* joint space narrowing

^a^Adjusted for age, sex (time-invariant), body mass index, rheumatoid arthritis duration, rheumatoid factor (time-invariant), DAS28-esr score, prednisone use, cardiac disorders, hypertension, osteoporosis, hand surgery, large joint osteoarthritis, or hip/knee arthroplasty

^b^NA: less than 5 outcomes not applicable for result estimation

### Incidence of osteoarthritis

A total of 894 patients without radiographic DIP OA at baseline were included in cohort 2. They were less likely to be female than those with OA. Patients without OA in the bDMARD group had lower mean BMI values than those in the csDMARD, combination, past user, and never-user groups (Additional file 1: Table S11). Proportions of rheumatoid factor positivity were around 30% in both the csDMARD and bDMARD groups. In time-varying Cox regression analysis, the incidence of DIP OA was not higher in the bDMARD group than in the csDMARD group (0.89 [95% CI 0.56–1.43]) (Table 5). In a subgroup analysis, neither osteoporosis nor prednisone therapy was associated with the incidence of radiographic DIP OA (Additional file 1: Table S12).

**Table 5:** Incidence rates, hazard ratios, and odds ratios of incident hand OA per treatment group following Cox regression analysis and GEE analysis among those without distal interphalangeal osteoarthritis at baseline (*N* = 894)

Exposure	Incident hand OA events	Person-time [years]	IR per 1000 person-years	Crude HR (95% CI)	Adjusted HR (95% CI) ^a^	Crude OR (95% CI)	Adjusted OR (95% CI)^a^
Analysis using baseline information only							
csDMARD	119	2239.0	53.1	Ref (1.00)	Ref (1.00)	NA	NA
bDMARD	10	147.2	67.9	1.44 (0.77–2.67)	1.41 (0.75–2.65)	NA	NA
Combination	43	750.6	57.3	1.13 (0.80–1.60)	1.09 (0.75–1.58)	NA	NA
Past-use	4	55.2	72.5	1.40 (0.54–3.61)	1.85 (0.73–4.66)	NA	NA
Never-use	22	793.1	27.7	0.52 (0.33–0.84)	0.59 (0.36–0.97)	NA	NA
Time-varying analysis							
csDMARD	96	1952.7	49.2	Ref (1.00)	Ref (1.00)	Ref (1.00)	Ref (1.00)
bDMARD	20	377.6	53	0.94 (0.59–1.50)	0.89 (0.56–1.43)	1.14 (0.70–1.87)	1.05 (0.63–1.75)
Combination	68	1212.7	56.1	1.06 (0.78–1.44)	1.07 (0.78–1.46)	1.08 (0.79–1.49)	1.04 (0.75–1.44)
Past-use	7	111.4	62.8	1.06 (0.51–2.19)	1.08 (0.50–2.33)	1.51 (0.68–3.32)	1.46 (0.66–3.25)
Never-use	7	330.7	21.2	0.55 (0.26–1.19)	0.72 (0.32–1.61)	0.92 (0.42–2.01)	1.05 (0.47–2.33)

*bDMARD* biologic disease-modifying antirheumatic drug, *CI* confidence interval, *csDMARD* conventional synthetic disease-modifying antirheumatic drug, *IR* incidence rate, *HR* hazard ratio

^a^Adjusted for age, sex (time-invariant), body mass index, rheumatoid arthritis duration, rheumatoid factor (time-invariant), DAS28-esr score, prednisone use, cardiac disorders, hypertension, osteoporosis, hand surgery, large joint osteoarthritis, or hip/knee arthroplasty

### Sensitivity analyses

Values of HRs of progression of DIP OA in bDMARD users compared to csDMARD users remained unchanged when not adjusting for DAS28-esr (1.35 [95% CI 1.07–1.70]) and slightly attenuated when leading the outcome by 6 months (1.24 [95% CI 0.97–1.59]) (Additional file 1: Tables S13–S14). HRs of incident DIP OA remained largely unchanged in sensitivity analyses when not adjusting for DAS28-esr or when leading the outcome by 6 months (Additional file 1: Tables S15–S16).

## Discussion

In this cohort study, among 2234 RA patients with at least two hand radiographs, followed for around 4 years, 1340 patients had radiographic DIP OA at baseline, and we observed a 34% increased risk of radiographic progression associated with bDMARD monotherapy. More specifically, this finding was mainly characterized by increased new bone formation. Among the 894 patients without DIP OA at baseline, we observed that the risk of incident radiographic OA did not differ between those receiving csDMARDs or bDMARDs, either in mono- or combination therapy.

This is a comprehensive analysis of both incident and pre-existing radiographic DIP OA including individual KLS components (i.e., osteophytes, joint space narrowing, sclerosis, and erosions) in a well-characterized RA patient registry database. We assessed the outcomes in both time-invariant and time-varying analyses, we adjusted for an extensive set of covariates to minimize residual confounding, and we conducted several sensitivity analyses to demonstrate the robustness of our findings. Furthermore, we used two-level multiple imputation to limit confounding by missingness. However, despite the strengths of this study, we must interpret our findings in light of several limitations. First, bDMARD monotherapy, the group of interest, was small and may be subject to selection bias as bDMARD monotherapy is not the preferred administration but cs/bDMARD combination therapy. In a previous study of the SCQM registry, initial bDMARD monotherapy was more often prescribed to more complex cases with older RA patients and higher rates of co-morbidity [16]. Similar results were obtained in our study where bDMARD users had the longest disease duration, highest age, and proportions of women and of patients with hypertension, all of them being risk factors to develop DIP OA [17]. While we did control our study population for aforementioned covariates, residual confounding may remain. Second, groups were not controlled for calendar time which may have resulted in time trend biases given a study period of 17 years. However, we controlled our analyses for factors which influence treatment choice (e.g., RA disease activity), and bDMARDs were available throughout the study period. Third, DAS28-esr scores at baseline in all exposure groups were relatively high; thus, our results might not be generalizable to RA patients in remission or low disease activity. Potentially, the high activity scores can be explained by a large enrollment of patients at the start of DMARD therapy courses and more radiographs being performed early in the ultrasound and pre-treat to target era. Fourth, since the outcomes could only be assessed when radiographs were taken, we likely missed the exact onset of DIP OA or DIP OA progression. However, DIP OA evolvement and progression is a slow progress, and in a sensitivity analysis when leading the outcome by 6 months, the results remained largely unchanged for incident DIP OA but were slightly attenuated in bDMARD users for DIP OA progression which may reveal residual confounding in this analysis but may also be due to decreased sample size. Unfortunately, information on DIP OA symptoms or joint function was not available, and the sample size did not allow for stratification by DIP OA severity at cohort entry DIP. Furthermore, we noted that patients at their first-hand radiographs differed from all RA patients identified in SCQM at their first visit with regard to higher proportions of DMARDs use, but a lower proportion of cardiac disorders, large joint OA, and previous hand surgery, and longer RA duration. Thus, our results may not be generalizable to the general RA population. Finally, the fact that radiographic scoring was performed by a single reader can be interpreted as a further weakness of this study.

The observed increased risk of DIP OA progression in bDMARD users was unexpected as previous trials investigating bDMARD monotherapy for the treatment of HOA (without RA) revealed either no or a small positive impact on structural progression [7, 9]. However, those studies only assessed smaller sample sizes versus non-use during a maximal 1-year follow-up, without analyzing individual KLS components. Our results are partially in contrast to the results of Loef et al. who showed that TNF-csDMARD combination was associated with reduced radiographic DIP OA progression up to 10 years follow-up in patients with concomitant RA [10]. A reduced incidence of DIP OA was also observed in RA patients under infliximab-csDMARD combination therapy but has never been studied for bDMARD monotherapy [4].

We postulate that the anti-erosive effects of bDMARDs, achieved by reducing osteoclastogenesis and the production of RANK ligand, are outweighed by these drugs’ known bone anabolic effects, for instance, the antagonism of Dkk-1, which finally can lead to increased osteophytosis [18]. This hypothesis is supported by the finding that osteophyte growth was the leading radiographic feature of increased progression under bDMARDs. We speculate that methotrexate has a more pleiotropic effect on other cell types, including osteoblasts, thus limiting the pro-osteogenic effects of bDMARDs on existing osteophytes [19]. Furthermore, our results suggest that bDMARD monotherapy is not restricted to the progression of osteophytes but also to joint space narrowing and potentially erosions (in small sample size). Clearly, central erosions in DIP OA are associated with joint space remodeling and differ from marginal erosions occurring in RA. Potentially, this finding is in line with the description of increased transition from the erosive into the remodeling phase under methotrexate therapy [6]. The hypothesis of a detrimental pro-osteogenic effect of DMARDs is supported to some extent by the finding that methotrexate and TNFi increase bone density in RA patients but do not reduce pathological new bone formation in with psoriatic arthritis and not or only discretely in spondylarthritis [20, 21].

Finally, our results suggest that concomitant osteoporosis and/or osteoporosis treatment reduced the progression of DIP OA. In the literature, lumbar bone mineral density is positively correlated with osteophytosis and subchondral sclerosis in hand OA but negatively with joint space narrowing or erosions [22]. Indeed, smaller previous studies have shown positive treatment effects of bisphosphonates such as clodronate on pain in hand OA [23]. However, the current literature is limited; therefore, we can only speculate if low bone density, or anti-osteoporosis therapy (e.g., bisphosphonates), is protective.

These results raise awareness of possible radiographic worsening of DIP OA in RA patients, especially those receiving bDMARD monotherapy. A baseline radiograph of the hand is therefore important to assess and monitor for RA lesions, but also for DIP OA. Ideally, a prospective study is needed to make recommendations for bDMARDs for RA patients suffering from radiographic of DIP OA.

## Conclusion

Biological DMARD monotherapy potentially enhances the risk of progression of pre-existing DIP OA in RA patients mainly as a result of increased osteophyte growth. Direct or indirect osteo-anabolic properties of targeted bDMARD therapy might be responsible for this observation, at least in this cohort of predominantly female and postmenopausal RA patients. Hand radiographs of RA patients at baseline should be assessed for distal HOA lesions. In patients with pre-existing DIP OA undergoing bDMARD monotherapy, hands should be monitored radiographically.

## Supplementary Information

**Additional file 1: Table S1.** Mean duration between visits in years and standard deviation. **Figure S1.** Patient examples of exposure evaluation for time-varying analyses. bDMARD: biologic disease modifying anti-rheumatic drug. csDMARD: conventional synthetic disease modifying anti-rheumatic drug. **Table S2.** Missingness per variable throughout follow-up. **Table S3.** Missingness pattern of variables with missingness only of Cohort 1 throughout follow-up (6136 observations). **Table S4.** Missingness pattern of variables with missingness only of Cohort 2 throughout follow-up (4511 observations). **Table S5.** Crude data table. **Table S6.** Data table for time-invariant Cox proportional hazard regression. **Table S7.** Data table for time-invariant Cox proportional hazard regression. **Table S8.** Patient characteristics of patients before exclusion versus patients with one or ≥2 radiographs. **Table S9.** Median duration between radiographs in years and interquartile range. **Table S10**. Results of Cox regression analyses of progression of DIP OA crude, fully adjusted, and when adjusting for fewer variables. **Table S11.** Patient characteristics of patient without osteoarthritis at cohort entry. **Table S12.** Hazard ratios of incident hand OA per treatment group following Cox time varying regression analyses in subgroups of age, rheumatoid factor, osteoporosis (treatment), and prednisone use. **Table S13.** Hazard ratios of hand OA progression per treatment group following time-varying Cox proportional hazard regression analyses when not adjusting for DAS28-esr. **Table S14.** Hazard ratios of hand OA progression per treatment group following time-varying Cox proportional hazard regression analyses when leading the outcome hand OA progression by 6 months. **Table 15.** Hazard ratios of hand OA incidence per treatment group following Cox time-varying proportional hazard regression analyses without adjusting for DAS28-esr. **Table 16.** Hazard ratios of hand OA incidence per treatment group following Cox time-varying regression analyses when leading the outcome hand OA progression by 6 months.

## Data Availability

Data are available upon reasonable request after having obtained approval from the data holder (SCQM).

## Abbreviations

RA: Rheumatoid arthritis
OA: Osteoarthritis
HOA: Hand osteoarthritis
csDMARDs: Conventional synthetic disease-modifying antirheumatic drugs
bDMARDs: Biological conventional synthetic disease-modifying antirheumatic drugs
DIP: Distal interphalangeal joint
DIP OA: Distal interphalangeal joint osteoarthritis
KLS: Modified Kellgren-Lawrence score
GEE: Generalized estimating equations
OR: Odds ratio
CI: Confidence interval
HR: Hazard ratio
TNFi: TNF inhibitors
SCQM: Swiss Clinical Quality Management in Rheumatic Diseases
MCP: Metacarpophalangeal joints
AIC: Akaike Information Criterion
PH: Proportional hazard
