# Supplementary material for: Increased radiographic progression of distal hand osteoarthritis occurring during biologic DMARD monotherapy for concomitant rheumatoid arthritis
Source: Arthritis Res Ther. 2021 Oct 26;23:267. doi: 10.1186/s13075-021-02654-0 (PMC8547039; doi:10.1186/s13075-021-02654-0)
Supplement: Supplementary file 1 — Additional file 1: Table S1. Mean duration between visits in years and standard deviation. Figure S1. Patient examples of exposure evaluation for time-varying analyses. bDMARD: biologic disease modifying anti-rheumatic drug. csDMARD: conventional synthetic disease modifying anti-rheumatic drug. Table S2. Missingness per variable throughout follow-up. Table S3. Missingness pattern of variables with missingness only of Cohort 1 throughout follow-up (6136 observations). Table S4. Missingness pattern of variables with missingness only of Cohort 2 throughout follow-up (4511 observations). Table S5. Crude data table. Table S6. Data table for time-invariant Cox proportional hazard regression. Table S7. Data table for time-invariant Cox proportional hazard regression. Table S8. Patient characteristics of patients before exclusion versus patients with one or ≥2 radiographs. Table S9. Median duration between radiographs in years and interquartile range. Table S10. Results of Cox regression analyses of progression of DIP OA crude, fully adjusted, and when adjusting for fewer variables. Table S11. Patient characteristics of patient without osteoarthritis at cohort entry. Table S12. Hazard ratios of incident hand OA per treatment group following Cox time varying regression analyses in subgroups of age, rheumatoid factor, osteoporosis (treatment), and prednisone use. Table S13. Hazard ratios of hand OA progression per treatment group following time-varying Cox proportional hazard regression analyses when not adjusting for DAS28-esr. Table S14. Hazard ratios of hand OA progression per treatment group following time-varying Cox proportional hazard regression analyses when leading the outcome hand OA progression by 6 months. Table 15. Hazard ratios of hand OA incidence per treatment group following Cox time-varying proportional hazard regression analyses without adjusting for DAS28-esr. Table 16. Hazard ratios of hand OA incidence per treatment group following Cox time-varying regre [file 13075_2021_2654_MOESM1_ESM.docx]

**Supplementary file DIP OA**

**Supplementary file 1:** Mean duration between visits in years and standard deviation

**Supplementary Table 1.** Mean duration between visits in years and standard deviation

|  | Mean duration between visits [years] (SD) | | | | |
| --- | --- | --- | --- | --- | --- |
|  | csDMARD | bDMARD | Combination therapy | Past DMARD use | Never DMARD use |
| Cohort 1^a^ | 0.8 (0.9) | 0.8 (1.0) | 0.8 (0.9) | 0.9 (0.9) | 1.5 (1.0) |
| Cohort 2^b^ | 0.9 (0.9) | 0.9 (0.9) | 0.8 (0.9) | 1.0 (1.0) | 1.8 (1.0) |

bDMARD: biologic disease modifying anti-rheumatic drug, csDMARD: conventional synthetic disease modifying anti-rheumatic drug, SD: standard deviation

^a^ assessing progression of hand OA

^b^ assessing incident hand OA

**Supplementary file 2:** Patient examples of exposure evaluation for time-varying analyses

Current exposure included 31 days after end of supply. Thus, past users were defined as having been off treatment for at least this period (see Patient A or B in Supplementary Figure 1). Treatment spells stopped and re-started within this period where connected as one current spell (see Patient C in Supplementary Figure 1).

Exposures of interest were assessed at cohort entry for time-invariant analyses only.

For time-varying analyses, exposure of interest was additionally assessed at every other radiograph or visit. A total of 50% of DMARD treatments were started on a radiograph/visit date, an additional 25% were started within 2.5 months prior to a radiograph/visit date. This adds up to 75% of DMARD treatments which were started within 2.5 months prior to a radiograph/visit date. Therefore, exposure was assumed to remain constant until the next radiograph/visit. Finally, in time-varying analyses, patients likely changed exposure groups during their observation period.


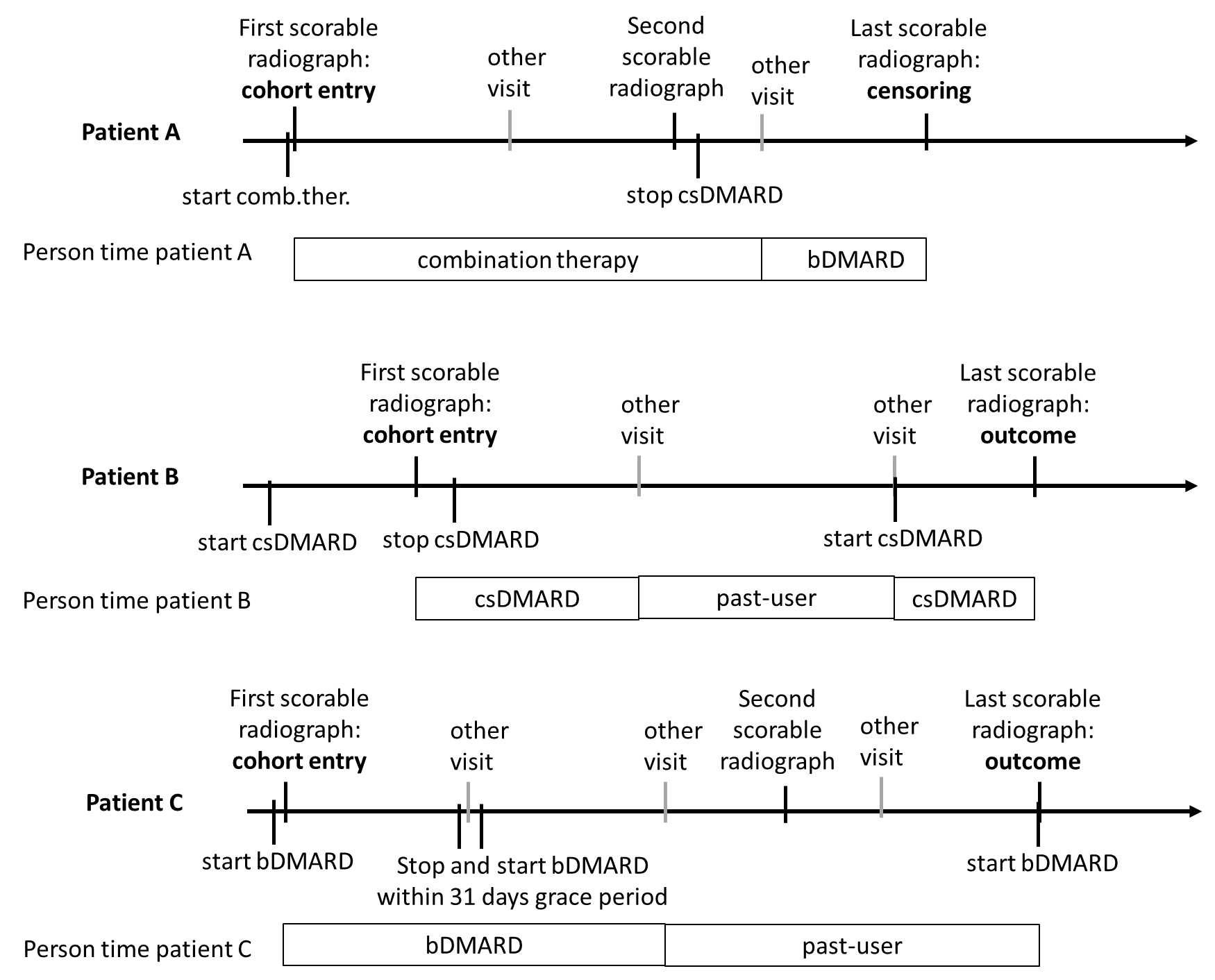


**Supplementary Figure 1.** Patient examples of exposure evaluation for time-varying analyses

bDMARD: biologic disease modifying anti-rheumatic drug

csDMARD: conventional synthetic disease modifying anti-rheumatic drug

**Supplementary file 3:** Detailed information on multiple imputation procedure

Before imputing the missing data we assessed overall missingness. Maximum missingness in a single variable was 8.1% of body mass index values in cohort 1 and 8.3% of DAS28-esr score values in cohort 2 (Supplementary Table2).

**Supplementary Table 2.** Missingness per variable throughout follow-up

|  | BMI | RF | DAS28-esr | RA duration |
| --- | --- | --- | --- | --- |
| Cohort 1^a^ | 8.1% | 2.5% | 6.0% | 2.2% |
| Cohort 2^b^ | 7.2% | 2.0% | 8.3% | 5.5% |

BMI: body mass index; RA: rheumatoid arthritis; RF: rheumatoid factor;

^a^ assessing progression of hand OA

^b^ assessing incident hand OA

When assessing missingness patterns, we observed that around 15% of patients had no missingness in cohort 1 (Supplementary Table3) and that around 20% had no missingness in cohort 2 (Supplementary Table4).

**Supplementary Table 3.** Missingness pattern of variables with missingness only of Cohort 1 throughout follow-up (6136 observations)

| **BMI** | **DAS28-esr** | **Rheumatoid Factor** | **RA duration** | **Frequence** | **Percent** |
| --- | --- | --- | --- | --- | --- |
|  |  |  |  |  |  |
| X | X | X | X | 5180 | 84.42 |
| X | X | X | . | 92 | 1.5 |
| X | X | . | X | 106 | 1.73 |
| X | X | . | . | 10 | 0.16 |
| X | . | X | X | 230 | 3.75 |
| X | . | X | . | 10 | 0.16 |
| X | . | . | X | 12 | 0.2 |
| X | . | . | . | 2 | 0.03 |
| . | X | X | X | 359 | 5.85 |
| . | X | X | . | 15 | 0.24 |
| . | X | . | X | 8 | 0.13 |
| . | . | X | X | 89 | 1.45 |
| . | . | X | . | 6 | 0.1 |
| . | . | . | X | 14 | 0.23 |
| . | . | . | . | 3 | 0.05 |

BMI: body mass index; RA: rheumatoid arthritis

**Supplementary Table 4.** Missingness pattern of variables with missingness only of Cohort 2 throughout follow-up (4511 observations)

| **BMI** | **DAS28-esr** | **Rheumatoid Factor** | **RA duration** | **Frequence** | **Percent** |
| --- | --- | --- | --- | --- | --- |
|  |  |  |  |  |  |
| X | X | X | X | 3645 | 80.8 |
| X | X | X | . | 188 | 4.17 |
| X | X | . | X | 64 | 1.42 |
| X | X | . | . | 12 | 0.27 |
| X | . | X | X | 250 | 5.54 |
| X | . | X | . | 18 | 0.4 |
| X | . | . | X | 7 | 0.16 |
| X | . | . | . | 1 | 0.02 |
| . | X | X | X | 219 | 4.85 |
| . | X | X | . | 6 | 0.13 |
| . | X | . | X | 1 | 0.02 |
| . | . | X | X | 70 | 1.55 |
| . | . | X | . | 24 | 0.53 |
| . | . | . | X | 5 | 0.11 |
| . | . | . | . | 1 | 0.02 |

BMI: body mass index; RA: rheumatoid arthritis;

We assumed that data is missing at random (MAR). Since we had longitudinal data with repeated observations per patient we used BLIMP software 2.2 which allows for multi-level imputation. We decided to use Fully conditional specification (FCS) because we had to impute values for 1 categorical and 3 continuous variables. Within FCS, we used the Gibbs sampler which avoids computational problems when some clusters have few observations (i.e. some patients had few observations). Covariates were modelled individually with individual distribution within FCS. Furthermore, the specification allowed for unequal cluster sizes. We requested common residual variance for all clusters and a Jeffrey (uniform) prior distribution was set for all regression coefficients in the imputation model.

For diagnostics of the multiple imputation model, we assessed the potential scale reduction (PSR) factor for different burn-in levels. PSR levels of <1.05 were achieved after a few hundred burn-in rounds. However, we used 2000 burn-ins to be at a PSR of <1.02. A PSR level of <1.05 indicates that the model converged.

In order to achieve convergence of the model, we had to simplify the model by deleting categorical variables with high missingness (e.g. activity level).

**Supplementary file 4:** Example of data management for each analysis: regular Cox proportional hazard regression model, Cox time-varying proportional hazard regression, and GEE analysis

The dataset needed to be manipulated to be fit for the different models we ran. Thus we want to share the following information.

Supplementary Table 5 shows the crude data table.

**Supplementary Table 5. Crude data table**

| patient | Age | Sex | OA | CVD | Other covariates | DAS28esr | Exposure | Visit date / radiograph date | Follow-up |
| --- | --- | --- | --- | --- | --- | --- | --- | --- | --- |
| 1 | 40 | 1 | 0 | 0 | … | 3.5 | 0 | 13.04.2000 | 0 |
| 1 | 41 | 1 | 0 | 1 | … | 4.0 | 1 | 24.04.2001 | 380 |
| 1 | 42 | 1 | 0 | 1 | … | 3.8 | 2 | 01.07.2002 | 790 |
| 1 | 43 | 1 | 1 | 1 | … | 2.9 | 2 | 02.02.2003 | 1000 |
| 2 | 68 | 0 | 0 | 0 | … | . | 3 | 25.06.2010 | 0 |
| 2 | 69 | 0 | 0 | 0 | … | . | 0 | 25.04.2011 | 300 |
| 2 | 70 | 0 | 0 | 1 | … | . | 3 | 26.05.2012 | 700 |

Blue lines represent the first eligible radiograph or other visit of a patient,

red lines the second last observation

green lines the last eligible radiograph or the outcome date.

Data table was manipulated to allow time-invariant Cox proportional hazard regression analysis. Only covariate information at cohort entry was used. Outcome information from the last eligible radiograph or outcome date was used and respective total follow-up was used.

**Supplementary Table 6. Data table for time-invariant Cox proportional hazard regression**

| patient | Age | Sex | OA | CVD | Other covariates | DAS28esr | Exposure | Visit date / radiograph date | Follow-up |
| --- | --- | --- | --- | --- | --- | --- | --- | --- | --- |
| 1 | 40 | 1 | 1 | 0 | … | 3.5 | 0 | 13.04.2000 | 1000 |
| 2 | 68 | 0 | 0 | 0 | … | . | 3 | 25.06.2010 | 700 |

In time-varying analyses, covariate information was assessed at the beginning of an interval. In other words, covariate information from the last eligible radiograph of a patient was never used, to prevent reverse causation. Supplementary table7 shows the data table used for GEE analysis (using start variable as time indicator) and Cox time-varying regression analysis (using start and stop variable to take into account time intervals)

**Supplementary Table 7. Data table for time-invariant Cox proportional hazard regression**

| patient | Age | Sex | OA | CVD | Other covariates | DAS28esr | Exposure | Visit date / radiograph date | Start | Stopp |
| --- | --- | --- | --- | --- | --- | --- | --- | --- | --- | --- |
| 1 | 40 | 1 | 0 | 0 | … | 3.5 | 0 | 13.04.2000 | 0 | 380 |
| 1 | 41 | 1 | 0 | 1 | … | 4.0 | 1 | 24.04.2001 | 380 | 790 |
| 1 | 42 | 1 | 1 | 1 | … | 3.8 | 2 | 01.07.2002 | 790 | 1000 |
| 2 | 68 | 0 | 0 | 0 | … | . | 3 | 25.06.2010 | 0 | 300 |
| 2 | 69 | 0 | 0 | 0 | … | . | 0 | 25.04.2011 | 300 | 700 |

**Supplementary file 5:** Patient characteristics before and after application of exclusion criteria

**Supplementary Table 8:** Patient characteristics of patients before exclusion versus patients with one or ≥2 radiographs.

| Patient characteristics | 8203 patients  at their first visit with SCQM | 2869 patients  at their first radiograph with only 1 radiograph | 2234 patients  at their first radiograph with at least 2 scorable radiographs |
| --- | --- | --- | --- |
| Mean Age [years] (SD) | 55.4 (13.7) | 56.5 (11.0) | 56.2 (11.1) |
| Female (%) | 6086 (74.2%) | 2179 (76.0%) | 1700 (76.1%) |
| Mean BMI (SD) | 25.5 (5.0) | 25.2 (4.7) | 25.3 (4.6) |
| Missing (%) | 1924 (0.2%) | 801 (0.3%) | 593 (0.3%) |
| Median RA duration [years] (IQR) | 4.7 (1.5-11.5) | 6.2 (2.2-13.4) | 6.0 (2.2-13.1) |
| Missing (%) | 782 (0.1%) | 130 (0.0%) | 85 (0.0%) |
| RF negative (%) | 1629 (19.9%) | 927 (32.3%) | 721 (32.3%) |
| RF positive (%) | 5097 (62.1%) | 1817 (63.3%) | 1432 (64.1%) |
| Missing (%) | 1477 (18.0%) | 125 (4.4%) | 81 (3.6%) |
| Mean DAS28-esr ^a^ (SD) | 4.2 (1.5) | 4.4 (1.5) | 4.4 (1.5) |
| Missing (%) | 1049 (0.0%) | 483 (0.0%) | 340 (0.0%) |
| ≥365 days of current csDMARD use ^b^ (%) | 1659 (20.2%) | 809 (28.2%) | 652 (29.2%) |
| ≥365 days of current bDMARD use ^c^ (%) | 369 (4.5%) | 165 (5.8%) | 129 (5.8%) |
| Prednisone use ^d^ (%) | 3778 (46.1%) | 1313 (45.8%) | 1031 (46.2%) |
| Cardiac disorders ^e^ (%) | 1286 (15.7%) | 201 (7.0%) | 152 (6.8%) |
| Hypertension ^f^ (%) | 1089 (13.3%) | 300 (10.5%) | 234 (10.5%) |
| Osteoporosis or fracture ^g^ (%) | 505 (6.2%) | 195 (6.8%) | 139 (6.2%) |
| Large joint osteoarthritis ^h^ (%) | 1035 (12.6%) | 226 (7.9%) | 157 (7.0%) |
| Hand surgery (%) | 1838 (22.4%) | 504 (17.6%) | 403 (18.0%) |

DIP OA: distal interphalangeal joint osteoarthritis; bDMARD: biologic disease-modifying antirheumatic drug; BMI: body mass index; csDMARD: conventional synthetic disease-modifying antirheumatic drug; DAS: disease activity score; esr: erythrocyte sedimentation rate; IQR: interquartile range; RA: rheumatoid arthritis; RF: rheumatoid factor; SCQM: Swiss Clinical Quality Management; SD: standard deviation;

^a^ DAS28-esr = (0.56×√[TJC28] + 0.28×√[SJC28] + 0.70×ln[ESR])×1.08 + 0.16

^b^ csDMARD use include methotrexate, leflunomid, sulfasalazin, chloroquine, azathioprine, cyclosporin A, cyclophosphamid,

^c^ bDMARD use include abatacept, adalimumab, anakinra, certolizumab, etanercept, golimumab, infliximab, rituximab, tocilizumab

^d^ Prednisone use include systemic or intra-articular prednisone use

^e^ Cardiac disorders include angina pectoris, myocardial infarction, arrhythmias, ischemic heart failure, angioplasty, or their treatment

^f^ Hypertension includes also anti-hypertensive treatment

^g^ Osteoporosis or fractures include also anti-osteoporotic treatment

^h^ Large joint osteoarthritis includes hip/knee replacements

**Supplementary file 6:** Median duration between radiographs in years and interquartile range

**Supplementary Table 9.** Median duration between radiographs in years and interquartile range

|  | Mean duration between radiographs [years] (IQR) | | | | |
| --- | --- | --- | --- | --- | --- |
|  | csDMARD | bDMARD | Combination therapy | Past DMARD use | Never DMARD use |
| Cohort 1^a^ | 3.0 (2.0-4.3) | 3.2 (2.1-4.4) | 3.1 (2.0-4.4) | 3.2 (2.2-4.6) | 2.4 (2.0-3.3) |
| Cohort 2^b^ | 3.2 (2.1-5.3) | 4.0 (2.2-6.2) | 3.7 (2.2-5.9) | 4.0 (2.1-5.2) | 2.4 (2.0-3.9) |

bDMARD: biologic disease modifying anti-rheumatic drug, csDMARD: conventional synthetic disease modifying anti-rheumatic drug, IQR: interquartile range

^a^ assessing progression of hand OA

^b^ assessing incident hand OA

**Supplementary file 7:** Additional results of Cox regression analyses of DIP OA progression when adjusting for fewer variables

**Supplementary Table 10:** Results of Cox regression analyses of progression of DIP OA crude, fully adjusted, and when adjusting for fewer variables

| Exposure | Hand OA progression events | Person-Time  [years] | IR per 1000 person-years | Crude HR  (95% CI) | Adjusted HR (95% CI) ^a^ | Adjusted HR (95% CI) ^b^ |
| --- | --- | --- | --- | --- | --- | --- |
| Analysis using baseline information only | | | | | |  |
| csDMARD | 526 | 3306.0 | 159.1 | Ref (1.00) | Ref (1.00) | Ref (1.00) |
| bDMARD | 34 | 244.7 | 138.9 | 0.96  (0.71-1.31) | 1.07  (0.78-1.46) | 1.05  (0.77-1.42) |
| Combination | 132 | 886.6 | 148.9 | 1.03  (0.85-1.24) | 1.09  (0.89-1.32) | 1.10  (0.91-1.34) |
| Past-use | 8 | 53.8 | 148.7 | 1.24  (0.70-2.19) | 1.15  (0.60-2.20) | 1.23  (0.67-2.27) |
| Never-use | 83 | 629.7 | 131.8 | 0.77  (0.61-0.97) | 0.81  (0.63-1.02) | 0.79  (0.63-1.00) |
| Time-varying Cox regression analysis | | | | |  |  |
| csDMARD | 421 | 2885.7 | 145.9 | Ref (1.00) | Ref (1.00) | Ref (1.00) |
| bDMARD | 79 | 391.9 | 201.6 | 1.23  (0.98-1.54) | 1.34  (1.07-1.69) | 1.35  (1.08-1.70) |
| TNFi | 67 | 357.6 | 187.4 | 1.16  (0.91-1.48) | 1.26  (0.98-1.62) | 1.27  (0.99-1.63) |
| Non-TNFi | 12 | 34.3 | 349.9 | 1.85  (1.18-2.89) | 2.07  (1.35-3.20) | 2.10  (1.36-3.25) |
| Combination | 236 | 1378.7 | 171.2 | 1.05  (0.90-1.22) | 1.12  (0.96-1.31) | 1.13  (0.97-1.32) |
| TNFi | 210 | 1293.7 | 162.3 | 1.01  (0.86-1.18) | 1.09  (0.92-1.28) | 1.09  (0.93-1.28) |
| Non-TNFi | 26 | 85 | 305.9 | 1.54  (1.10-2.15) | 1.56  (1.10-2.23) | 1.58  (1.11-2.26) |
| Past-use | 24 | 128.4 | 186.9 | 0.96  (0.64-1.42) | 0.96  (0.66-1.41) | 0.98  (0.67-1.42) |
| Never-use | 23 | 335.9 | 68.5 | 0.51  (0.31-0.83) | 0.54  (0.33-0.90) | 0.52  (0.31-0.87) |

bDMARD: biologic disease-modifying antirheumatic drug; CI: confidence interval; csDMARD: conventional synthetic disease-modifying antirheumatic drug; IR: incidence rate; HR: hazard ratio; OA: osteoarthritis; OR: odds ratio; TNFi: tumor necrosis factor inhibitor

^a^ adjusted for age, sex (time invariant), body mass index, rheumatoid arthritis duration, rheumatoid factor (time invariant), DAS28-esr score, prednisone use, cardiac disorders, hypertension, osteoporosis, hand surgery, large joint osteoarthritis or hip/knee arthroplasty

^b^ adjusted for age, sex (time invariant), rheumatoid arthritis duration, hypertension, osteoporosis

**Supplementary file 8:** Patient characteristics of patient without osteoarthritis at cohort entry

**Supplementary Table 11.** Patient characteristics of patient without osteoarthritis at cohort entry

| Patient characteristics  at baseline visit | csDMARD  n=496 | bDMARD  n=44 | Combination  n=196 | Past-use  n=15 | Never-use  n=143 |
| --- | --- | --- | --- | --- | --- |
| Mean Age [years] (SD) | 50.5 (10.0) | 50.8 (10.7) | 51.5 (9.6) | 47.4 (7.9) | 49.8 (11.0) |
| Female (%) | 384 (77.4%) | 32 (72.7%) | 135 (68.9%) | 13 (86.7%) | 103 (72.0%) |
| Mean Follow-up time [years] (SD) | 4.5 (2.7) | 3.3 (1.8) | 3.8 (2.3) | 3.7 (2.3) | 5.5 (3.9) |
| Mean BMI (SD) | 24.6 (4.6) | 23.1 (3.0) | 25.4 (4.6) | 25.7 (4.1) | 24.9 (5.0) |
| Missing (%) | 82 (16.5%) | 1 (2.3%) | 7 (3.6%) | 4 (26.7%) | 76 (53.1%) |
| Median RA duration [years] (IQR) | 4.7 (1.8-11.2) | 7.2 (4.0-13.6) | 8.2 (4.1-13.5) | 10.4 (7.1-18.1) | 3.0 (1.1-7.7) |
| Missing (%) | 14 (2.8%) | 1 (2.3%) | 3 (1.5%) | 1 (6.7%) | 26 (18.2%) |
| RA factor Negative (%) | 152 (30.7%) | 13 (29.6%) | 70 (35.7%) | 5 (33.3%) | 37 (25.9%) |
| RA factor Positive (%) | 333 (67.1%) | 28 (63.6%) | 121 (61.7%) | 6 (40.0%) | 98 (68.5%) |
| Missing (%) | 11 (2.2%) | 3 (6.8%) | 5 (2.6%) | 4 (26.7%) | 8 (5.6%) |
| Mean DAS28-esr ^a^ (SD) | 3.9 (1.5) | 4.2 (1.6) | 4.0 (1.5) | 4.8 (1.3) | 4.6 (1.5) |
| Missing (%) | 45 (9.1%) | 6 (13.6%) | 27 (13.8%) | 5 (33.3%) | 68 (47.6%) |
| ≥365 days of current csDMARD use ^b^ (%) | 148 (29.8%) | 0 | 94 (48.0%) | 0 | 0 |
| ≥365 days of current bDMARD use ^c^ (%) | 0 | 12 (27.3%) | 43 (21.9%) | 0 | 0 |
| Prednisone use ^d^ (%) | 235 (47.4%) | 16 (36.4%) | 108 (55.1%) | 6 (40.0%) | 32 (22.4%) |
| Cardiac disorders ^e^ (%) | 23 (4.6%) | 1 (2.3%) | 21 (10.7%) | 1 (6.7%) | 7 (4.9%) |
| Hypertension ^f^ (%) | 78 (15.7%) | 6 (13.6%) | 43 (21.9%) | 1 (6.7%) | 13 (9.1%) |
| Osteoporosis or fracture ^g^ (%) | 40 (8.1%) | 6 (13.6%) | 41 (20.9%) | 2 (13.3%) | 6 (4.2%) |
| Large joint osteoarthritis ^h^ (%) | 23 (4.6%) | 5 (11.4%) | 22 (11.2%) | 0 | 5 (3.5%) |
| Hand surgery (%) | 38 (7.7%) | 7 (15.9%) | 16 (8.2%) | 1 (7.1%) | 2 (1.4%) |

bDMARD: biologic disease-modifying antirheumatic drug; BMI: body mass index; csDMARD: conventional synthetic disease-modifying antirheumatic drug; DAS: disease activity score; esr: erythrocyte sedimentation rate; IQR: interquartile range; RA: rheumatoid arthritis; SD: standard deviation;

^a^ DAS28-esr = (0.56×√[TJC28] + 0.28×√[SJC28] + 0.70×ln[ESR])×1.08 + 0.16

^b^ csDMARD use include methotrexate, leflunomid, sulfasalazin, chloroquine, azathioprine, cyclosporin A, cyclophosphamid,

^c^ bDMARD use include abatacept, adalimumab, anakinra, certolizumab, etanercept, golimumab, infliximab, rituximab, tocilizumab

^d^ Prednisone use include systemic or intra-articular prednisone use

^e^ Cardiac disorders include angina pectoris, myocardial infarction, arrhythmias, ischemic heart failure, angioplasty, or their treatment

^f^ Hypertension includes also anti-hypertensive treatment

^g^ Osteoporosis or fractures include also anti-osteoporotic treatment

^h^ Large joint osteoarthritis includes hip/knee replacements

**Supplementary file 9. Hazard ratios of incident hand OA per treatment group following Cox time‑varying regression analyses**

**Supplementary Table 12.** Hazard ratios of incident hand OA per treatment group following Cox time‑varying regression analyses in subgroups of age, rheumatoid factor, osteoporosis (treatment), and prednisone use

| Exposure | Incident hand OA events | Person-Time  [years] | Crude HR  (95% CI) | Adjusted HR  (95% CI) ^a^ |
| --- | --- | --- | --- | --- |
| Age ≤ 55 years at baseline | | | | |
| csDMARD | 63 | 1360.9 | Ref (1.00) | Ref (1.00) |
| bDMARD | 12 | 292.2 | 0.76 (0.41-1.38) | 0.74 (0.40-1.40) |
| Combination | 40 | 881.9 | 0.89 (0.60-1.33) | 0.89 (0.59-1.35) |
| Past-use | 5 | 88.6 | 1.05 (0.46-2.40) | 1.07 (0.46-2.52) |
| Never-use | 4 | 251.5 | 0.43 (0.16-1.15) | 0.63 (0.22-1.80) |
| Age ˃ 55 years at baseline |  |  |  |  |
| csDMARD | 33 | 591.8 | Ref (1.00) | Ref (1.00) |
| bDMARD | 8 | 85.5 | 1.51 (0.75-3.04) | 1.50 (0.75-3.02) |
| Combination | 28 | 330.8 | 1.45 (0.89-2.37) | 1.31 (0.79-2.17) |
| Past-use | 2 | 22.9 | 1.14 (0.24-5.42) | 1.14 (0.22-6.04) |
| Never-use | 3 | 79.2 | 0.93 (0.26-3.28) | 0.84 (0.21-3.32) |
| Rheumatoid factor negative at baseline |  |  |  |  |
| csDMARD | 35 | 623.9 | Ref (1.00) | Ref (1.00) |
| bDMARD | 5 | 150.8 | 0.53 (0.21-1.35) | 0.51 (0.2-1.27) |
| Combination | 29 | 441.5 | 1.16 (0.71-1.88) | 1.14 (0.69-1.89) |
| Past-use | 2 | 28.9 | 1.05 (0.23-4.76) | 1.45 (0.29-7.26) |
| Never-use | 3 | 109.4 | 0.57 (0.19-1.70) | 0.78 (0.23-2.62) |
| Rheumatoid factor positive at baseline |  |  |  |  |
| csDMARD | 58 | 1291 | Ref (1.00) | Ref (1.00) |
| bDMARD | 14 | 209.9 | 1.27 (0.73-2.18) | 1.19 (0.69-2.07) |
| Combination | 39 | 742.4 | 1.07 (0.72-1.60) | 1.10 (0.73-1.65) |
| Past-use | 5 | 78.8 | 1.06 (0.46-2.45) | 0.96 (0.39-2.37) |
| Never-use | 4 | 208.6 | 0.57 (0.20-1.63) | 0.74 (0.25-2.18) |
| Osteoporosis/ fracture or osteoporosis treatment (time-varying) | | | | |
| csDMARD without osteoporosis | 79 | 1680.6 | Ref (1.00) | Ref (1.00) |
| csDMARD with osteoporosis | 17 | 272.1 | 1.16 (0.68-1.99) | 0.86 (0.50-1.48) |
| bDMARD without osteoporosis | 14 | 274.2 | 0.93 (0.54-1.60) | 0.89 (0.51-1.56) |
| bDMARD with osteoporosis | 6 | 103.4 | 1.06 (0.47-2.38) | 0.77 (0.34-1.71) |
| Combination therapy without osteoporosis | 52 | 970.3 | 1.04 (0.73-1.47) | 1.04 (0.73-1.48) |
| Combination therapy with osteoporosis | 16 | 242.3 | 1.30 (0.77-2.18) | 0.99 (0.56-1.76) |
| Never/past-use without osteoporosis | 14 | 413.1 | 0.80 (0.46-1.40) | 0.95 (0.53-1.69) |
| Never/past-use with osteoporosis | 0 | 29.1 | NA | NA |
| Prednisone treatment (time-varying) | | | | |
| csDMARD without prednisone | 45 | 1098.2 | 0.87 (0.47-1.60) | 0.85 (0.44-1.61) |
| csDMARD with prednisone | 51 | 854.5 | 1.35 (0.73-2.47) | 1.45 (0.76-2.78) |
| bDMARD without prednisone | 12 | 220.8 | Ref (1.00) | Ref (1.00) |
| bDMARD with prednisone | 8 | 156.9 | 1.01 (0.43-2.34) | 0.93 (0.39-2.23) |
| Combination therapy without prednisone | 39 | 639.0 | 1.19 (0.64-2.22) | 1.27 (0.66-2.42) |
| Combination therapy with prednisone | 29 | 573.7 | 1.06 (0.55-2.04) | 1.07 (0.53-2.16) |
| Never/past-use without prednisone | 9 | 345.1 | 0.63 (0.27-1.45) | 0.74 (0.31-1.79) |
| Never/past-use with prednisone | 5 | 97.1 | 1.33 (0.46-3.84) | 1.50 (0.52-4.34) |

bDMARD: biologic disease-modifying antirheumatic drug; CI: confidence interval; csDMARD: conventional synthetic disease-modifying antirheumatic drug; HR: hazard ratio

^a^ adjusted for age, sex (time invariant), body mass index, rheumatoid arthritis duration, rheumatoid factor (time invariant), DAS28-esr score, prednisone use, cardiac disorders, hypertension, osteoporosis, hand surgery, large joint osteoarthritis or hip/knee arthroplasty

**Supplementary file 10:** Hazard ratios of hand OA progression per treatment group in time-varying Cox proportional hazard regression analyses when not adjusting for DAS28-esr or when leading the outcome by 6 months (183 days)

**Supplementary Table 13.** Hazard ratios of hand OA progression per treatment group following time-varying Cox proportional hazard regression analyses when not adjusting for DAS28-esr

| Exposure | Hand OA progression events | Person-Time  [years] | Crude HR  (95% CI) | Adjusted HR ^a^  (95% CI) | Adjusted HR ^a^  (95% CI) without DAS28-esr |
| --- | --- | --- | --- | --- | --- |
| csDMARD | 421 | 2885.7 | Ref (1.00) | Ref (1.00) | Ref (1.00) |
| bDMARD | 79 | 391.9 | 1.23 (0.98-1.54) | 1.34 (1.07-1.69) | 1.35 (1.07-1.70) |
| TNFi | 67 | 357.6 | 1.16 (0.91-1.48) | 1.26 (0.98-1.62) | 1.27 (0.99-1.63) |
| Non-TNFi | 12 | 34.3 | 1.85 (1.18-2.89) | 2.07 (1.35-3.20) | 2.09 (1.36-3.21) |
| Combination | 236 | 1378.7 | 1.05 (0.90-1.22) | 1.12 (0.96-1.31) | 1.12 (0.96-1.31) |
| TNFi | 210 | 1293.7 | 1.01 (0.86-1.18) | 1.09 (0.92-1.28) | 1.09 (0.92-1.28) |
| Non-TNFi | 26 | 85 | 1.54 (1.10-2.15) | 1.56 (1.10-2.23) | 1.56 (1.10-2.23) |
| Past-use | 24 | 128.4 | 0.96 (0.64-1.42) | 0.96 (0.66-1.41) | 0.96 (0.66-1.40) |
| Never-use | 23 | 335.9 | 0.51 (0.31-0.83) | 0.54 (0.33-0.90) | 0.54 (0.33-0.90) |

bDMARD: biologic disease-modifying antirheumatic drug; CI: confidence interval; csDMARD: conventional synthetic disease-modifying antirheumatic drug; HR: hazard ratio; non-TNFi : non- tumor necrosis factor inhibitors; OA: osteoarthritis; TNFi : tumor necrosis factor inhibitor;

^a^ adjusted for age, sex (time invariant), body mass index, rheumatoid arthritis duration, rheumatoid factor (time invariant), DAS28-esr score, prednisone use, cardiac disorders, hypertension, osteoporosis, hand surgery, large joint osteoarthritis or hip/knee arthroplasty

**Supplementary Table 15.** Hazard ratios of hand OA progression per treatment group following time-varying Cox proportional hazard regression analyses when leading the outcome hand OA progression by 6 months

| Exposure | Hand OA progression events | Person-Time  [years] | Crude HR  (95% CI) | Adjusted HR ^a^  (95% CI) |
| --- | --- | --- | --- | --- |
| csDMARD | 425 | 2673.2 | Ref (1.00) | Ref (1.00) |
| bDMARD | 73 | 353.7 | 1.16 (0.91-1.47) | 1.24 (0.97-1.59) |
| TNFi | 63 | 325 | 1.10 (0.85-1.42) | 1.18 (0.90-1.54) |
| Non-TNFi | 10 | 28.7 | 1.75 (1.08-2.85) | 1.86 (1.15-3.01) |
| Combination | 238 | 1260.7 | 1.08 (0.92-1.27) | 1.13 (0.96-1.33) |
| TNFi | 215 | 1188.1 | 1.04 (0.89-1.23) | 1.10 (0.93-1.30) |
| Non-TNFi | 23 | 72.6 | 1.65 (1.11-2.45) | 1.68 (1.12-2.53) |
| Past-use | 24 | 116.5 | 1.05 (0.69-1.59) | 1.05 (0.70-1.59) |
| Never-use | 23 | 324.4 | 0.48 (0.30-0.78) | 0.52 (0.32-0.84) |

bDMARD: biologic disease-modifying antirheumatic drug; CI: confidence interval; csDMARD: conventional synthetic disease-modifying antirheumatic drug; HR: hazard ratio; OA: osteoarthritis; non-TNFi : non- tumor necrosis factor inhibitors; TNFi : tumor necrosis factor inhibitor;

^a^ adjusted for age, sex (time invariant), body mass index, rheumatoid arthritis duration, rheumatoid factor (time invariant), DAS28-esr score, prednisone use, cardiac disorders, hypertension, osteoporosis, hand surgery, large joint osteoarthritis or hip/knee arthroplasty

**Supplementary file 11:** Hazard ratios of incident hand OA per treatment group in time-varying Cox proportional hazard regression analyses when not adjusting for DAS28-esr or when leading the outcome by 6 months (183 days)

**Supplementary Table 15.** Hazard ratios of hand OA incidence per treatment group following Cox time-varying proportional hazard regression analyses without adjusting for DAS28-esr

| Exposure | Incident hand OA events | Person-Time  [years] | Crude HR  (95% CI) | Adjusted HR ^a^  (95% CI) | Adjusted HR ^a^  (95% CI) without DAS28-esr |
| --- | --- | --- | --- | --- | --- |
| csDMARD | 96 | 1952.7 | Ref (1.00) | Ref (1.00) | Ref (1.00) |
| bDMARD | 20 | 377.6 | 0.94 (0.59-1.50) | 0.89 (0.56-1.43) | 0.90 (0.56-1.44) |
| Combination | 68 | 1212.7 | 1.06 (0.78-1.44) | 1.07 (0.78-1.46) | 1.06 (0.78-1.46) |
| Past-use | 7 | 111.4 | 1.06 (0.51-2.19) | 1.08 (0.50-2.33) | 1.05 (0.49-2.24) |
| Never-use | 7 | 330.7 | 0.55 (0.26-1.19) | 0.72 (0.32-1.61) | 0.67 (0.30-1.47) |

bDMARD: biologic disease-modifying antirheumatic drug; CI: confidence interval; csDMARD: conventional synthetic disease-modifying antirheumatic drug; HR: hazard ratio; OA: osteoarthritis;

^a^ adjusted for age, sex (time invariant), body mass index, rheumatoid arthritis duration, rheumatoid factor (time invariant), DAS28-esr score, prednisone use, cardiac disorders, hypertension, osteoporosis, hand surgery, large joint osteoarthritis or hip/knee arthroplasty

**Supplementary Table 16.** Hazard ratios of hand OA incidence per treatment group following Cox time-varying regression analyses when leading the outcome hand OA progression by 6 months

| Exposure | Incident hand OA events | Person-Time  [years] | Crude HR  (95% CI) | Adjusted HR ^a^  (95% CI) |
| --- | --- | --- | --- | --- |
| csDMARD | 98 | 1859.6 | Ref (1.00) | Ref (1.00) |
| bDMARD | 20 | 357.2 | 1.00 (0.61-1.65) | 1.00 (0.61-1.64) |
| Combination | 60 | 1142.6 | 1.00 (0.72-1.39) | 1.00 (0.72-1.39) |
| Past-use | 5 | 105.3 | 0.91 (0.38-2.20) | 0.92 (0.38-2.24) |
| Never-use | 10 | 323.5 | 0.75 (0.38-1.49) | 0.77 (0.39-1.53) |

bDMARD: biologic disease-modifying antirheumatic drug; CI: confidence interval; csDMARD: conventional synthetic disease-modifying antirheumatic drug; HR: hazard ratio; OA: osteoarthritis;

^a^ adjusted for age, sex (time invariant), body mass index, rheumatoid arthritis duration, rheumatoid factor (time invariant), DAS28-esr score, prednisone use, cardiac disorders, hypertension, osteoporosis, hand surgery, large joint osteoarthritis or hip/knee arthroplasty
